# A structural equation modelling analysis: interprofessional team collaboration, organizational career management, and post competency of community nurses

**DOI:** 10.1186/s12913-023-09303-z

**Published:** 2023-04-01

**Authors:** Li Ma, Xinwei Wang, Shiyue Zou, Min Lin, Shi Qiu, Weimin Li

**Affiliations:** 1 Institute of Hospital Management, Outpatient Department, West China School of Nursing, West China Hospital, Sichuan University, Chengdu, Sichuan China; 2 School of Business, Sichuan University, Chengdu, Sichuan China; 3 The First People’s Hospital of Longquanyi District, Chengdu, Sichuan China; 4 West China Biomedical Big Data Center, West China Hospital, Sichuan University, Chengdu, Sichuan China; 5 Department of Respiratory and Critical Care Medicine, Institute of Respiratory Health, Frontiers Science Center for Disease-Related Molecular Network/Precision Medicine Research Center, West China Hospital, Sichuan University, Chengdu, Sichuan China

**Keywords:** Structural equation model, Community nurse, Post competency, Interprofessional team collaboration, Organizational career management

## Abstract

**Background:**

With the advent of an ageing society and an increase in the prevalence of chronic diseases, the role of primary health care has become increasingly important and reliant on multidisciplinary collaboration. As members of this interprofessional cooperative team, community nurses play a dominant role. Thus, the post competencies of community nurses study deserve our attention. In addition, organizational career management can affect nurses in some ways. This study aims to examine the current situation and relationship among interprofessional team collaboration, organizational career management and post-competency of community nurses.

**Methods:**

A survey was conducted among 530 nurses in 28 community medical institutions from November 2021 to April 2022 in Chengdu, Sichuan Province, China. Descriptive analysis was used for analysis, and a structural equation model was used to hypothesize and verify the model. A total of 88.2% of respondents met the inclusion criteria and did not meet the exclusion criteria. The main reason nurses gave for not participating was that they were too busy.

**Results:**

Among the competencies on the questionnaire, ensuring quality and helping roles scored the lowest. The teaching-coaching and diagnostic functions played a mediating role. Nurses with greater seniority and those who were transferred to administrative departments had lower scores, and the difference was statistically significant (*p* < 0.05). In the structural equation model, CFI = 0.992 and RMSEA = 0.049, which shows that the model fit well, suggesting that organizational career management had no statistically significant effect on post competency (β = -0.006, *p* = 0.932) but that interprofessional team collaboration had a statistically significant effect on post competency (β = 1.146, *p* < 0.001) and organizational career management had a statistically significant effect on interprofessional team collaboration (β = 0.684, *p* < 0.001).

**Conclusions:**

Attention should be given to the improvement of community nurses' post competency in ensuring quality and performing helping, teaching-coaching, and diagnostic roles. Moreover, researchers should focus on the decline in community nurses' abilities, particularly for those with greater seniority or in administrative roles. The structural equation model shows that interprofessional team collaboration is a complete intermediary between organizational career management and post competency.

## Background

The ageing population, chronic disease prevalence, the ubiquity of COVID, and financial restructuring have increased people's demand for health services, showing multilevel and diversified characteristics [1, 2]. Nurses, as the core force in the field of primary health care, need to have sufficient ability to cope with increasingly complex and onerous nursing work to meet people's health needs [3, 4]. Research shows that nurses’ competency at their jobs, or post competency (PC), is the core embodiment of their ability to perform their jobs and ensures the provision of high-quality nursing service [5, 6], which is closely related to the quality of medical care, nurses' work efficiency, and patient satisfaction. It is these abilities on which nursing education should focus [7]. Another study defines post competency as a series of personality characteristics that can distinguish between excellent and ordinary job performance and as a combination of knowledge, skills, values, and so on [8]. Post competency among nurses refers to the sum of personal characteristics, knowledge, and skills that enable them to be competent in their job performance and provide excellent medical care.

In the modern health care system, the provision of medical services has changed from independent practice to a cross-disciplinary team-based approach, which involves multiple professionals with different educational backgrounds, training and professional knowledge seeking a common goal [9, 10]. Lack of collaboration among health professionals will significantly increase the possibility of providing poor health care, making drug and surgical errors that may contribute to patient deaths and increasing staff turnover in a complex medical environment [11–14]. Improving interprofessional team collaboration has received considerable attention, as it is a key factor in improving the efficiency of medical care [15, 16]. Interprofessional team collaboration (ITC) refers to a cooperative mode in which two or more medical professionals and their clients participate, cooperate, coordinate and make common decisions on health and social issues [17, 18]. Interprofessional team collaboration is considered a cost-effective medical practice method to address this situation, and its importance has been recognized by various organizations and individuals [19–21]. However, in clinical practice, due to certain communication problems among medical staff, differences in the sense of cooperation, change in partners, unfamiliarity with each other's abilities, and other reasons [22], interprofessional team collaboration cannot achieve the ideal state and is thus unable to provide high-quality medical services.

It is very important to understand the impact of hospital organizational factors and the nursing delivery process on medical safety [23]. Organizational career management (OCM) refers to a series of management methods implemented by the organization that aim to develop the potential of employees, retain employees, and enable employees to realize their career goals. Career management research has obtained some valuable results [24–28]. Long et al. [29] stated that if an organization attaches importance to employees' career management, it can fully develop employees' human resources to improve their quality and retain these trained employees who can actively contribute to the development of the organization, achieving a win–win for both. These studies also show that organizational career management is of great importance to personal development [30–32]. Therefore, organizational career management may affect the post competency of community nurses.

A literature review showed that the research subjects related to nurses' post competency are nurses in relatively important departments (e.g., operating room, emergency department, paediatrics), junior nurses and nursing students in large general hospitals [33]. Research reports on the status quo of the post competency of community nurses engaged in primary health care are relatively rare. However, the role of community nurses in health care cannot be underestimated. Moreover, nurses' interprofessional team collaborative ability is very important to the quality of the primary health care services they provide [34–37]. In addition, organizational career management provided by community medical institutions may affect nurses’ post competency through psychology and behaviour [29, 38].

The purpose of this study is to use a structural equation model (SEM) to assume and verify the relationships among interprofessional team collaboration, organizational career management and post competency and to analyse the influencing factors so that we can recommend corresponding interventions to improve primary health care.

## Methods

### Hypothesis model

**Fig. 1:**
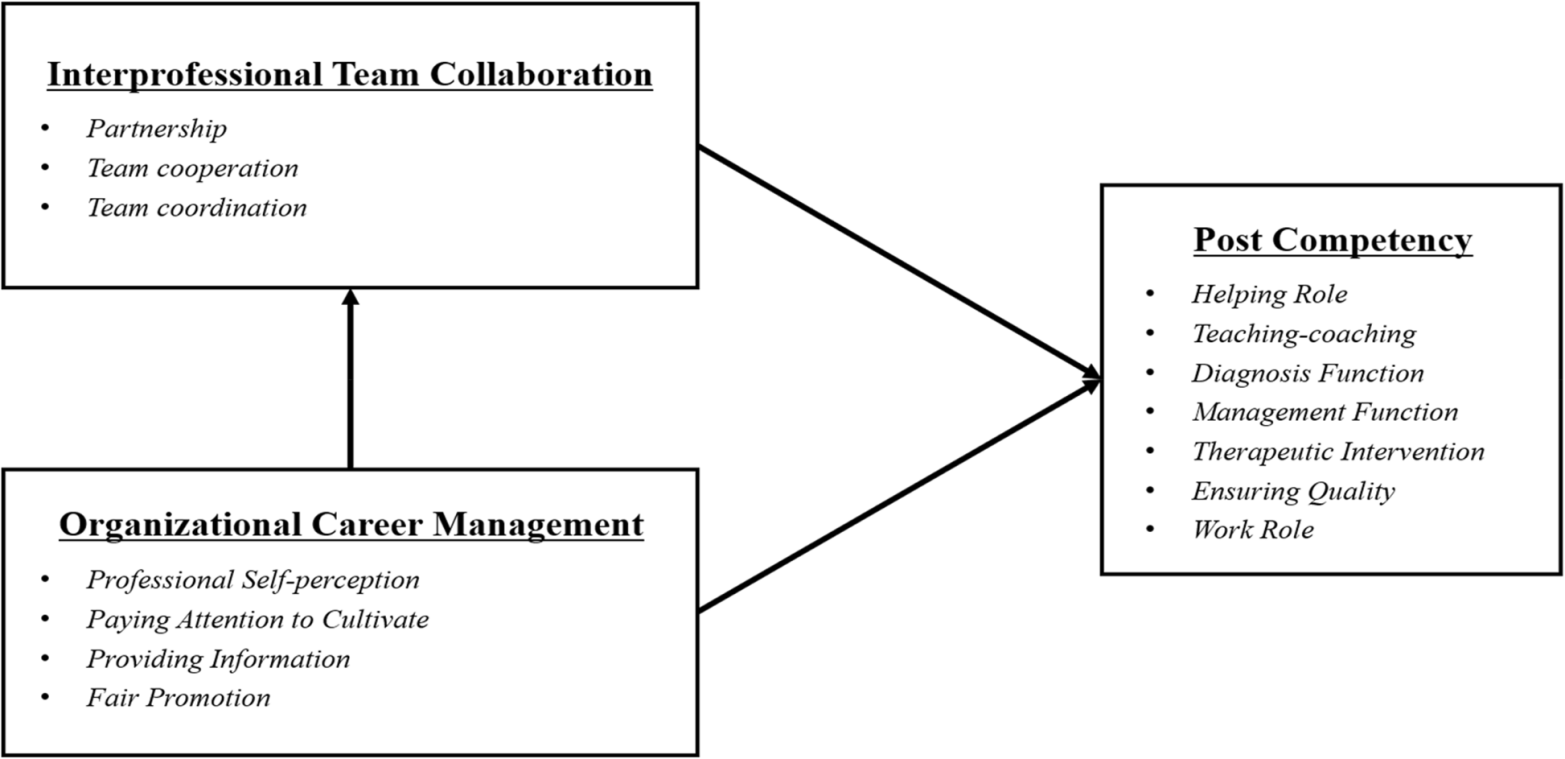
Hypothesis model

In this paper, we mainly explore the impact of interprofessional team collaboration and organizational career management on the post competency of community nurses. Studies have shown that nurses with better ITC and OCM may have more robust PC [19–25]. Moreover, nurses with better OCM may have stronger ITC [19, 25]. Therefore, the hypothetical model we set up is shown in Fig. 1, and we made the following hypotheses:

- H1: Community nurses’ interprofessional team collaboration is positively correlated with their post competency.
- H2: The organizational career management of community nurses is positively correlated with their post competency.

- H3: The organizational career management of community nurses is positively correlated with their interprofessional team collaboration .

### Participants and procedures

We designed questionnaires aimed at interprofessional team collaborative ability, organizational career management and post competency of community nurses. The questionnaire used in this study has 14 dimensions, so the sample size was calculated using the dimensions of the scale. The sample size = [Max (number of dimensions) × 20] x (1 + 15%) [39], and the sample size should be no less than 322. The survey was administered after the respondents agreed to participate in this study. We provided online written informed consent at the beginning of the online questionnaire and distributed links to community nurses in Chengdu, Sichuan Province, China. We collected a total of 530 valid questionnaires from 28 community medical institutions during the period from November 2021 to April 2022. The inclusion criteria were as follows: Registered nurses who had worked in community medical institutions for more than 1 year and volunteered to participate in this study were eligible. The exclusion criteria were as follows: Staff who were not willing to participate in this study were not included. To reduce bias, the study adopted the double-blind method for both the persons who issued the questionnaires and the persons who completed the questionnaires. The specific characteristics of the nurses we investigated can be seen in Table 1.

**Table 1:** Basic characteristics of respondents

Characteristics	Frequency	Percentage
Gender		
Female	509	96.04%
Male	21	3.96%
Educational background		
Technical secondary school	20	3.77%
Junior college	225	42.45%
Undergraduate	285	53.77%
Seniority		
1–2 years	53	10.00%
3–5 years	132	24.91%
6–9 years	148	27.92%
≥ 10 years	197	37.17%
Department		
Outpatient Department	237	44.7%
Inpatient Department	259	48.9%
Administration Department	34	6.4%

### Measures

The questionnaire we designed includes four parts. The first part asks for basic information about the participants, and the second part is the nurse post competency scale. The third part is the scale of interprofessional team collaboration, and the fourth part is the scale of organizational career management. The first part of the questionnaire mainly collected the basic information about the respondents, such as gender, professional title, education background, working years, working organization and working department. These data can provide feedback on respondents' basic data distribution and the survey scope.

Our investigation and evaluation of the competency of community nurses mainly referred to the nurse competency scale proposed by researchers [40]. We translated the scale into Chinese and made cross-cultural adjustments to meet the competency requirements of Chinese nurses. In this part, we mainly asked community nurses to evaluate their behaviour in daily nursing practice, with a score of 0 to 100 points for each item in this aspect. In addition, according to the actual situation, we evaluated the frequency of clinical practice activities, which was divided into four grades: never, rarely, occasionally and often. We quantified the competency of community nurses in seven aspects: helping role, teaching-coaching, diagnostic function, management function, therapeutic intervention, ensuring quality and work role. The helping role mainly refers to nurses specifying nursing plans according to people's needs, supporting their strategies, helping them adapt to the environment and making nursing decisions in accordance with professional ethical codes. Teaching-coaching means that nurses can carefully analyse the needs of people, plan rehabilitation education activities, guide nursing students and take measures to maintain and improve their professional skills. The diagnostic function means that nurses can analyse people's health status from multiple perspectives, such as physiology, psychology, and society, and guide other nurses to observe illness and use equipment. Management functions refer to the ability of nurses to identify and deal with situations that pose a threat to people at an early stage, to respond to changes in clinical situations and to keep equipment running. Therapeutic intervention refers to the ability of nurses to flexibly designate nursing activity plans according to specific clinical conditions and to support and promote the development of clinical pathways. Ensuring quality means that nurses can abide by nursing concepts, continuously improve nursing quality, and systematically evaluate their satisfaction with nursing. The work role refers to the ability of nurses to identify the support and help they need at the same time, to be familiar with the division of responsibilities and writing of nursing work and to ensure the smooth progress of the work.

To assess community nurses' interprofessional team collaborative ability, we relied on the Interprofessional Team Collaboration Scale proposed by researchers [41] and the Chinese translation of the scale [42]. The questions in this part describe the direct collaboration between nurses and colleagues of different professions, such as doctors, in their daily work. A total of 23 items are divided into three parts—partnership, team cooperation and coordination—each of which uses the Likert 5-point scoring method, a scale of 1 to 5 meaning never, rarely, sometimes, often, and always. Partnerships involve team members maintaining communication, discussions, and adjusting treatment and care plans with patients and their families, and all team members are engaged in treatment and goal setting. Team cooperation values team members' rights of sharing, mutual respect, and trust. Team coordination includes the ability to adjust team leaders as people need change, team members to recognize and implement treatment and care conflict resolution processes, and so on.

Based on the research of Long et al. [29], we constructed a community nurse organizational career management scale that reflected the organizational career management of community nurses from four aspects: professional self-perception, paying attention to cultivation, providing information, and fair promotion. Each item is assessed on a 4-point Likert scale, where 1–4 indicate very much disagree, somewhat disagree, somewhat agree, and very much agree, respectively. Among them, professional self-perception includes activities by the organization to understand its professional characteristics, plans to change jobs and performance feedback. The organization helps to plan and choose a career development path. Paying attention to cultivation includes reimbursement of academic education funds by the organization, conducting training, providing learning opportunities and materials, and estimating on-the-job training. The provided information consists of the promotion route information, job vacancy information, job qualification information and guidance work provided by the organization. Fair promotion includes promoting individuals according to personal ability and work performance in an acceptable competitive environment.

### Data analysis

We conducted statistical comparisons and Bonferroni tests on the post competency, interprofessional team collaboration and organizational career management scores of 530 community nurses with different demographic characteristics. The details are shown in Table 2.

**Table 2:** Scores of each dimension and Bonferroni test results under different demographic characteristics

Characteristics	Post Competency		Interprofessional Team Collaboration		Organizational Career Management	
	score	*t/F, P*	score	*t/F, P*	score	*t/F, P*
**Gender**						
Female	80.65 ± 20.82	*t* = -0.739,*P* = 0.460	93.29 ± 20.76	*t* = -0.598,*P* = 0.556	54.14 ± 9.12	*t* = 0.355,*P* = 0.722
Male	83.30 ± 15.89		96.03 ± 15.40		53.44 ± 8.89	
**Educational background**						
Technical secondary school	88.01 ± 11.20	*F* = 1.086, *P* = 0.338	102.00 ± 14.12	*F* = 1.589, *P* = 0.205	56.15 ± 8.69	*F* = 2.240,*P* = 0.107
Junior college	83.45 ± 17.17		95.56 ± 16.67		54.07 ± 9.16	
Undergraduate	82.65 ± 15.48		95.78 ± 14.84		52.80 ± 8.66	
**Seniority(years)**						
1 ~ 2	88.63 ± 14.30	*F* = 2.996,*P* = 0.030	102.74 ± 15.69	*F* = 4.682, *P* = 0.003	57.53 ± 7.90	*F* = 4.579,*P* = 0.004
3 ~ 5	83.17 ± 15.22		95.97 ± 14.77		53.42 ± 8.87	
6 ~ 9	83.75 ± 15.35		96.25 ± 16.40		53.35 ± 8.96	
≥ 10	81.32 ± 17.36		93.80 ± 15.15		52.49 ± 8.88	
**Department**						
Outpatient department	85.21 ± 14.91	*F* = 7.635, *P* = 0.001	98.21 ± 14.19	*F* = 8.090,*P* = 0.000	54.60 ± 8.25	*F* = 4.716,*P* = 0.009
Inpatient Department	82.54 ± 16.09		94.91 ± 16.38		52.84 ± 9.42	
Administration Department	74.14 ± 20.54		87.62 ± 16.18		50.35 ± 8.11	

In this study, we used IBM Statistical Package for Social Sciences (SPSS) and AMOS (Analysis of Moment Structure) version 28 to perform statistical and structural equation modelling analyses on the collected data. The mean, standard deviation, etc., are used to describe the demographic characteristics of the data. The correlations among organizational career management, interprofessional team collaborative ability, and the post competency of community nurses are analysed using covariance-based structural equation modelling. We employ maximum likelihood estimation and path analysis to determine their relationship. We use the ratio of chi-square degrees of freedom, root mean square residual (RMR), root mean square error of approximation (RMSEA), comparative fit index (CFI), goodness-of-fit index (GFI), normed fit index (NFI), and other indicators to evaluate the fit of the model. We believe the model fit is better, and the analysis results can be accepted in the following cases. For example, the ratio of chi-square degrees of freedom is between 1 and 3, both RMR and RMSEA are less than 0.5, and GFI, CFI, and NFI are greater than 0.9.

## Results

### Variable and correlation analysis

According to the questionnaire design, we set interprofessional team collaboration, organizational career management and post competency as the latent variables of the study. Helping role, teaching-coaching, diagnostic function, management function, therapeutic intervention, ensuring quality and work role are post competency variables. Partnership, team cooperation and team coordination are the observation variables corresponding to interprofessional team collaboration. Professional self-perception, paying attention to cultivation, providing information and fair promotion are the observed variables corresponding to organizational career management.

Since the score index calculation of each part of the questionnaire was inconsistent, we calculated the mean value of the items completed by community nurses under each observation variable and normalized the data. Then, we calculate the correlation between the observed variables, and the correlation matrix is shown in Table 3. We find that teaching-coaching and diagnostic functions, diagnostic functions and professional self-perception, partnership and team coordination, and team cooperation and team coordination all have strong correlations. The correlations between helping roles and work roles, ensuring quality, therapeutic intervention, and management functions are weaker.

**Table 3:** Correlations of Study Variables

	helping role	teaching-coaching	diagnosis function	management function	therapeutic intervention	ensuring quality	work role	partnership	team cooperation	team coordination	professional self-perception	paying attention to cultivate	providing information	fair promotion
helping role	1	-	-	-	-	-	-	-	-	-	-	-	-	-
teaching-coaching	0.878	1	-	-	-	-	-	-	-	-	-	-	-	-
diagnosis function	0.821	0.936	1	-	-	-	-	-	-	-	-	-	-	-
management function	0.354	0.405	0.414	1	-	-	-	-	-	-	-	-	-	-
therapeutic intervention	0.356	0.407	0.416	0.843	1	-	-	-	-	-	-	-	-	-
ensuring quality	0.331	0.378	0.387	0.784	0.865	1	-	-	-	-	-	-	-	-
work role	0.357	0.408	0.418	0.81	0.807	0.791	1	-	-	-	-	-	-	-
partnership	0.774	0.865	0.879	0.428	0.43	0.4	0.432	1	-	-	-	-	-	-
team cooperation	0.754	0.863	0.882	0.433	0.436	0.405	0.437	0.913	1	-	-	-	-	-
team coordination	0.772	0.883	0.903	0.444	0.446	0.414	0.448	0.934	0.945	1	-	-	-	-
professional self-perception	0.775	0.885	0.924	0.419	0.421	0.391	0.423	0.918	0.871	0.914	1	-	-	-
paying attention to cultivate	0.512	0.585	0.598	0.557	0.529	0.521	0.562	0.619	0.627	0.641	0.606	1	-	-
providing information	0.479	0.548	0.561	0.522	0.525	0.488	0.527	0.639	0.587	0.601	0.609	0.752	1	-
fair promotion	0.532	0.608	0.622	0.579	0.582	0.541	0.584	0.644	0.651	0.667	0.629	0.834	0.781	1

### Measurement model

We performed factor analysis on the variables in the measurement model. The Kaiser‒Meyer‒Olkin (KMO) value obtained by the KMO and Bartlett sphericity tests was 0.93, and the significance of the Bartlett sphericity test was less than 0.001, indicating that our data were suitable for factor analysis. The specific analysis results are shown in Table 4. The factor loadings of the observed variables we calculated ranged from 0.767 to 0.928, all of which were greater than 0.7, indicating statistical significance. In addition, we also carried out factor rotation analysis of the maximum variance method. After shielding the value less than 0.5, we found that the observed variables could be divided into three groups corresponding to the latent variables, which verified the feasibility of the measurement model we established.

**Table 4:** Confirmatory factor analysis

Indicators	Mean	Standard Deviation	Factor Loadings	PC	ITC	OCM
PC ≤ helping role	79.9547	17.80302	0.767	0.823		
PC ≤ teaching-coaching	83.0090	16.71791	0.914	0.896		
PC ≤ diagnosis function	83.1509	17.14130	0.928	0.908		
PC ≤ management function	85.2710	16.25431	0.912	0.897		
PC ≤ therapeutic intervention	82.6455	17.99393	0.923	0.898		
PC ≤ ensuring quality	82.1893	18.50187	0.884	0.875		
PC ≤ work role	84.2831	16.09339	0.912	0.891		
ITC ≤ partnership	4.1170	.72862	0.897		0.871	
ITC ≤ team cooperation	4.3245	.65443	0.813		0.744	
ITC ≤ team coordination	4.0553	.83127	0.859		0.768	
OCM ≤ professional self-perception	3.2585	.64042	0.837			0.856
OCM ≤ paying attention to cultivate	3.3623	.58546	0.869			0.893
OCM ≤ providing information	3.3981	.57372	0.897			0.891
OCM ≤ fair promotion	3.3476	.59616	0.854			0.864

*PC* post competency, *ITC* interprofessional team collaboration, *OCM* organizational career management

### Parameter estimation and verification analysis

We built the structural equation model based on the hypothesis model and used Amos for verification analysis. In addition, we also modified the relationship between model observation variables and residual errors based on fitting results and model modification suggestions, and the model fitting index results are shown in Table 4. The ratio of the chi-square to the degrees of freedom of model fitting was 2.28, the RMSEA value was 0.049, and the CFI value was 0.992 (Table 5). These fitting coefficient values indicated that the model had good fitting performance and accurately reflected the relationship between the variables explored to a certain extent.

**Table 5:** Global fitting coefficients

	${X}^{2}/df$	RMR	RMSEA	GFI	CFI	NFI
Evaluation standard	< 3 and > 1	< 0.05	< 0.05	> 0.9	> 0.9	> 0.9
Inspection results	2.28	0.023	0.049	0.964	0.992	0.987

**Fig. 2:**
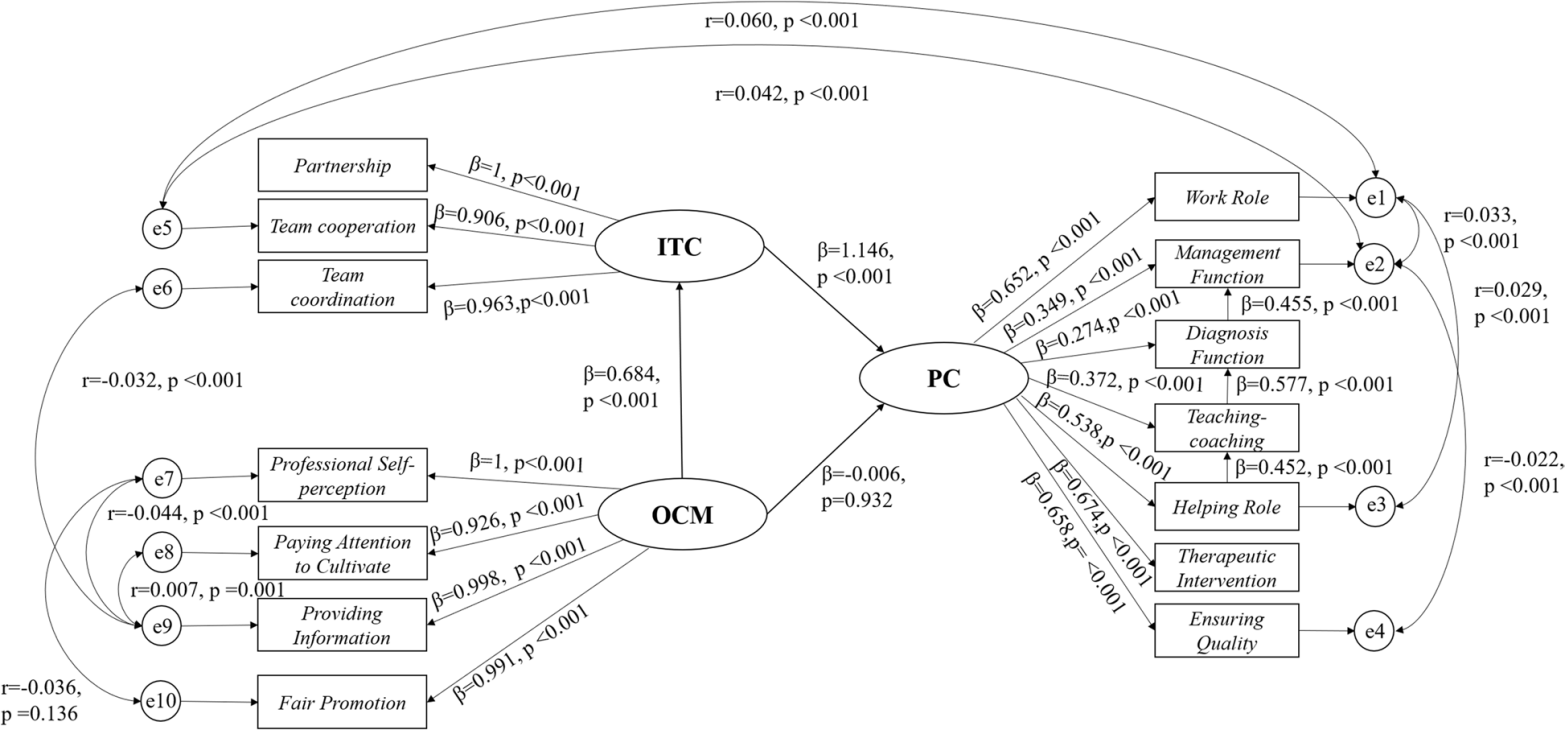
Model fitting results and path coefficients. PC = post competency, ITC = interprofessional team collaboration, OCM = organizational career management

Amos was used to fit the structural equation model, and the results are shown in Fig. 2. We found that the organizational career management of community nurses had no statistically significant effect on post competency (β = -0.006, *p* = 0.932), so H2 was rejected. However, community nurses' interprofessional team collaborative ability had a statistically significant and positive effect on their post competency (β = 1.146, *p* < 0.001), so we accept H1. Moreover, organizational career management among community nurses had a statistically significant impact on interprofessional team collaboration. Community nurses' organizational career management positively affected their interprofessional team collaborative behaviour (β = 0.684, *p* < 0.001), so we accept H3. Therefore, we can also judge that community nurses' interprofessional team collaborative ability completely mediates organizational career management ability and post competency. In addition, we also found that the helping role had a positive and significant impact on teaching-coaching (β = 0.452, *p* < 0.001), teaching/coaching on the diagnostic function (β = 0.577, *p* < 0.001), and the diagnostic function on the management function (β = 0.455, *p* < 0.001).

## Discussion

The scores of community nurses' PC in this study were as follows: management function (85.28 ± 16.25), work role (84.28 ± 16.09), diagnostic function (83.15 ± 17.14), teaching-coaching (83.02 ± 16.72), therapeutic intervention (82.65 ± 17.99), ensuring quality (82.19 ± 18.50) and helping role (79.95 ± 17.80). This study is consistent with the survey data of Faraji et al. [43] in the top ranking. Therefore, management functions are the best function of all, both in our study and in Faraji's study.

In this study, the management function and work role of community nurses were the two highest scoring dimensions, and ensuring quality and helping roles were the lowest. This may be related to community nurses in China undertaking a large share of public health and preventive health services (e.g., infectious disease management, chronic disease management, vaccination). This can help the management functions and work roles do better than others while neglecting the role of ensuring quality and helping role, which is closely related to medical safety and satisfaction. However, the study indicates that medical safety is a major concern in hospitals and primary health institutions and needs more effort to improve safety culture [44].

The helping role of community nurses has a significant positive effect on teaching-coaching and teaching-coaching to the diagnostic function and then the diagnostic function to the management function. This shows that teaching-coaching is the complete intermediary of the helping role to the diagnostic function. The diagnostic function is the complete intermediary between teaching-coaching and the management function. Nurses are found to be most suitable for carrying out tasks concerning health education [45]. Therefore, improving the teaching-coaching role is helpful to enhance the diagnosis directly and the management function indirectly, which can give direction to managers seeking to support community nurses.

In this study, there were significant differences in the evaluation of organizational career management, interprofessional team collaboration and post competency among community nurses with different seniorities and departments. Interestingly, the results of our study show that the greater the seniority is, the lower the self-evaluation results. In addition, the self-evaluation of community nurses who work in administrative departments is worse than that of nurses in inpatient and outpatient departments. This may be because the human resources of other professions in community medical institutions are relatively inadequate [46]. Nurses with greater seniority are often transferred from clinical nursing work to routine administration. As a result, there is a decline in the post competency of these nurses. When nurses with greater seniority mainly engaged in routine work, such as health statistics, the opportunities for interprofessional team collaboration decreased, so the ability of interprofessional team collaboration gradually decreased. Moreover, because community nurses who had more seniority or worked in administrative mainly engaged in auxiliary work rather than clinical work, their importance in community medical institutions might be ignored. Therefore, their opportunities, such as information feedback, training guidance and fair promotion, are also reduced. Organizational career management for senior or administrative department nurses is not as good as that for inpatient or outpatient department or junior nurses. Other studies have shown that junior nurses generally overestimate their post competency level, and junior evaluators tend to evaluate their competency levels higher than do senior evaluators [47–49]. Other studies have shown that nursing increasingly relies on information technology, and the social media use profile benefits both social skills and nurse–patient interaction [50, 51]. Junior nurses may be more familiar with information technology than senior nurses. These findings can explain why junior nurses had higher self-scoring results than senior nurses and why the scores of community nurses in the inpatient and outpatient departments were higher than those in the administrative department.

Defensive strategies to minimize some difficulties include the support of hierarchical superiors and the empowerment of the community in primary health care [38]. Therefore, organizational career management should have a significant positive impact on the post competency of nurses, despite the lack of government incentives [45]. Community medical institutions can improve the post competency of community nurses through professional self-perception, attention to cultivation, provision of information, and fair promotion. However, interestingly, after the verification of our model, the effect was not significant, which indicates that community medical institutions may, because of the shortage of human resources or for the sake of economic benefits [52], let nurses perform the work of other members in some places [46]. This practice ignores the organizational career management of community nurses by not providing them with relevant information about their careers and ignoring the opportunity for fair promotion. In addition, the shortage of human resources and heavy daily work [2, 46, 53] forces community nurses to remain at the same career level for a long time. It is also not conducive to the continuous improvement of the post competency of community nurses, which can explain our results.

The ability of nurses requires changes in the education and continuity of their qualifications [54], and organizational career management significantly impacts nurses' ability through psychodynamics [38]. Some scholars believe that organizational career management improves interprofessional team collaboration through education and training [2]. The ability of interprofessional team collaboration focuses on partnership, teamwork, and team coordination, which are closely related to communication and mutual respect [55]. Community medical institution managers believe that nurses play an important role in interprofessional team cooperation [34, 56], so organizational career management emphasizes cultivating the interprofessional team collaborative ability. Our study also validates these points.

Disease prevention and management are important tasks in community medical institutions. Team practices are gradually replacing individual practices; the management of many diseases can rely not only on a single person but also on more cross-disciplinary teamwork [45, 57]. In a team formed by doctors, nurses, pharmacists and managers, nurses are the core force and even leaders of the team [36, 37, 52, 58], and the interprofessional team collaborative ability of nurses is directly related to success or failure. Interprofessional team collaboration is important to achieve high-quality care and positive patient outcomes [59]. Therefore, interprofessional team collaborative ability significantly impacts nurses’ post competency, and our study validates this finding.

Strategies to ensure patient safety should focus on building leadership ability that supports a blame-free environment, open communication and continuous organizational learning [60]. In this study's confirmatory structural equation model, the direct impact of organizational career management on community nurses' post competency was not significant. However, the impact on interprofessional team collaboration was significant. In contrast, the impact of interprofessional team collaboration on post competency was significant. Hence, our study validates that organizational career management can affect post competency through interprofessional team collaboration.

## Limitations

The limitation of this study is that only community nurses in Chengdu, China, were selected. In addition, this study carried out relevant analysis, discussion, and judgement only through the self-assessment results of community nurses, which was somewhat subjective. In the future, we will extend this study to other cities and increase the sample size to make the results more generalizable. At the same time, we will try to include multiangle evaluations of community nurses from other perspectives, such as those of doctors, patients, and managers.

## Conclusions

Attention should be given to the improvement of community nurses' post competencies, such as ensuring quality, providing help, teaching and coaching, and diagnostic functions. Moreover, senior nurses or nurses transferred to administrative departments should focus on improving their abilities. The structural equation model shows that interprofessional team collaboration is a complete intermediary between organizational career management and post competency, which can contribute to health services research by improving community nurses' abilities.

## Data Availability

The main data generated or analysed during this study are included in this published article, and others are available from the corresponding author on reasonable request.

## Abbreviations

PC: Post competency
ITC: Interprofessional team collaboration
OCM: Organizational career management
SEM: Structural Equation Model
SPSS: Statistical Package for Social Sciences
AMOS: Analysis of Moment Structure
RMR: Root Mean Square Residual
RMSEA: Root Mean Square Error of Approximation
CFI: Comparative Fit Index
GFI: Goodness-of-Fit Index
NFI: Normed Fit Index
KMO: Kaiser‒Meyer‒Olkin value

## References

[1] Zhao F, Ahmed F, Faraz NA (2020). Caring for the caregiver during COVID-19 outbreak: does inclusive leadership improve psychological safety and curb psychological distress? a cross-sectional study. Int J Nurs Stud.

[2] Watkins S, Neubrander J (2020). Registered nurse education in primary care: barriers and resolutions. Nurs Forum.

[3] Lunde L, Moen A, Rosvold EO (2018). Learning clinical assessment and interdisciplinary team collaboration in primary care. MOOC for healthcare practitioners and students. Stud Health Technol Inform.

[4] Goltz HH, Major JE, Goffney J, Dunn MW, Latini D (2021). Collaboration between oncology social workers and nurses: a patient-centered interdisciplinary model of bladder cancer care. Semin Oncol Nurs.

[5] Smith SA (2012). Nurse competence: a concept analysis. Int J Nurs Knowl.

[6] Funk KA, Weaver KK (2018). Team work and collaborative practice agreements among pharmacists and nurse practitioners. J Am Pharm Assoc.

[7] Gardulf A, Nilsson J, Florin J, Leksell J, Lepp M, Lindholm C (2016). The nurse professional competence (NPC) scale: self-reported competence among nursing students on the point of graduation. Nurse Educ Today.

[8] Fitzpatrick R (1994). Competence at work: models for superior performance. Pers Psychol.

[9] Palanisamy R, Taskin N, Verville J (2017). Impact of trust and technology on interprofessional collaboration in healthcare settings: an empirical analysis. Int J e-Collab.

[10] Höglund B, Larsson M (2019). Midwives’ work and attitudes towards contraceptive counselling and contraception among women with intellectual disability: focus group interviews in Sweden. Eur J Contracept Reprod Health Care.

[11] Hadi-Moghaddam M, Karimollahi M, Aghamohammadi M (2021). Nurses’ trust in managers and its relationship with nurses’ performance behaviors: a descriptive-correlational study. BMC Nurs.

[12] Lee CY, Goeman D, Beanland C, Elliott RA (2019). Challenges and barriers associated with medication management for home nursing clients in Australia: a qualitative study combining the perspectives of community nurses, community pharmacists and GPs. Fam Pract.

[13] Norful AA, de Jacq K, Carlino R, Poghosyan L (2018). Nurse practitioner–physician comanagement: a theoretical model to alleviate primary care strain. Ann Fam Med.

[14] Redley B, Botti M, Wood B, Bucknall T (2017). Interprofessional communication supporting clinical handover in emergency departments: an observation study. Australas Emerg Nurs J.

[15] Leach LS, Myrtle RC, Weaver FA (2011). Surgical teams: role perspectives and role dynamics in the operating room. Health Serv Manag Res.

[16] Kilpatrick K, Tchouaket E, Fernandez N, Jabbour M, Dubois C-A, Paquette L (2021). Patient and family views of team functioning in primary healthcare teams with nurse practitioners: a survey of patient-reported experience and outcomes. BMC Fam Pract.

[17] Angela J, Marjorie L (2014). The continued need for interprofessional collaboration and research. Appl Nurs Res.

[18] Sullivan M, Kiovsky RD, Mason DJ, Hill CD, Dukes C (2015). Interprofessional collaboration and education. Am J Nurs.

[19] Ohta R, Ryu Y, Katsube T, Sano C (2020). Rural homecare nurses’ challenges in providing seamless patient care in rural Japan. Int J Environ Res Public Health.

[20] Oliveira IBd, Peres  AM, Martins MM, Bernardino  E , Haddad  MDCFL, Lowen  IMV (2021). Innovative actions developed by nurses in primary health care. Rev Bras Enferm.

[21] Vest JR, Caine V, Harris LE, Watson DP, Menachemi N, Halverson P (2018). Fostering local health department and health system collaboration through case conferences for at-risk and vulnerable populations. Am J Public Health.

[22] Van der Biezen M, Wensing M, Poghosyan L, van der Burgt R, Laurant M (2017). Collaboration in teams with nurse practitioners and general practitioners during out-of-hours and implications for patient care; a qualitative study. BMC Health Serv Res.

[23] Liu X, Zheng J, Liu K, Baggs JG, Liu J, Wu Y (2018). Hospital nursing organizational factors, nursing care left undone, and nurse burnout as predictors of patient safety: a structural equation modeling analysis. Int J Nurs Stud.

[24] Hill RE (1987). "Career development in organizations", by DT hall & associates. Hum Resour Manag.

[25] Pazy A (1988). Joint responsibility: the relationships between organizational and individual career management and the effectiveness of careers. Group Organ Stud.

[26] Iles P, Mabey C (1993). Managerial career development programmes: effectiveness, availability and acceptability. Br J Manag.

[27] Herriot P, Gibbons P, Pemberton C, Jackson PR (1994). An empirical model of managerial careers in organizations. Br J Manag.

[28] Crabtree MJ (1999). Employees' perceptions of career management practices: the development of a new measure. J Career Assess.

[29] Long L, Fang L, Ling W (2002). Organizational career management: measurement and its effects on employees' behaviour and feeling in China. Acta Psychol Sin.

[30] Güney S, Karadağ A, El-Masri M (2021). Perceptions and experiences of person-centered care among nurses and nurse aides in long term residential care facilities: a systematic review of qualitative studies. Geriatr Nurs.

[31] Lukewich J, Allard M, Ashley L, Aubrey-Bassler K, Bryant-Lukosius D, Klassen T (2020). National competencies for registered nurses in primary care: a Delphi study. West J Nurs Res.

[32] Owens RA (2021). Exploring family nurse practitioner professional identity formation at rural health care facilities. J Am Assoc Nurse Pract.

[33] Flinkman M, Leino-Kilpi H, Numminen O, Jeon Y, Kuokkanen L, Meretoja R (2017). Nurse competence scale: a systematic and psychometric review. J Adv Nurs.

[34] Adamakidou T, Triantafyllopoulou MN, Feleki P, Papadopoulou L, Kalokairinou A (2020). Team members' roles in home healthcare: evidence from the "AKEΣΩ-1" project in Greece. Home Healthc Now.

[35] Mororó DDS, Menezes RMP, Queiroz AAR, Silva CJA, Pereira WC (2020). Nurse as an integrator in healthcare management of children with chronic condition. Rev Bras Enferm.

[36] Selleck CS, Fifolt M, Burkart H, Frank JS, Curry WA, Hites LS (2017). Providing primary care using an interprofessional collaborative practice model: what clinicians have learned. J Prof Nurs.

[37] Wilson EC, Pammett R, McKenzie F, Bourque H (2021). Engagement of nurse practitioners in primary health care in northern British Columbia: a mixed-methods study. CMAJ Open.

[38] Rocha GSA, Silva D, Andrade MS, Andrade BBF, Medeiros SEG, Aquino JM (2021). Suffering and defense mechanisms: an analysis of the work of primary health care nurses. Rev Bras Enferm.

[39] Zou ZY, Liu RR, Zhai J, Zhang XT, Du J, Xu CP (2019). Correlation analysis of the competency level of new nurses in the grade a general hospitals of Jinan City. Occup Health.

[40] Meretoja R, Isoaho H, Leino-Kilpi H (2004). Nurse competence scale: development and psychometric testing. J Adv Nurs.

[41] Orchard C, Pederson LL, Read E, Mahler C, Laschinger H (2018). Assessment of interprofessional team collaboration scale (AITCS): further testing and instrument revision. J Contin Educ Health Prof.

[42] Cui J, Chen X, Lao Y, Zhuang Y (2019). Chinesization on simplified Assessment of Interprofessional Team Collaboration Scale and analysis on its reliability and validity. J Nurs Rehabil.

[43] Faraji A, Karimi M, Azizi SM, Janatolmakan M, Khatony A (2019). Evaluation of clinical competence and its related factors among ICU nurses in Kermanshah-Iran: a cross-sectional study. Int J Nurs Sci.

[44] Khamaiseh A, Al-Twalbeh D, Al-Ajlouni K (2020). Patient safety culture in Jordanian primary health-care centres as perceived by nurses: a cross-sectional study. East Mediterr Health J.

[45] Matthys E, Remmen R, Van Bogaert P (2019). Practice nurse support and task suitability in a general practice: a cross-sectional survey in Belgium. J Interprof Care.

[46] Torrens C, Campbell P, Hoskins G, Strachan H, Wells M, Cunningham M (2020). Barriers and facilitators to the implementation of the advanced nurse practitioner role in primary care settings: a scoping review. Int J Nurs Stud.

[47] Bahreini M, Moattari M, Ahmadi F, Kaveh MH, Hayatdavoudy P, Mirzaei M (2011). Comparison of head nurses and practicing nurses in nurse competence assessment. Iran J Nurs Midwifery Res.

[48] Meretoja R, Leino-Kilpi H (2003). Comparison of competence assessments made by nurse managers and practising nurses. J Nurs Manag.

[49] O'connor S, Pearce J, Smith R, Voegeli D, Walton P (2001). An evaluation of the clinical performance of newly qualified nurses: a competency based assessment. Nurse Educ Today.

[50] Rowett KE, Christensen D (2020). Oncology nurse navigation: expansion of the navigator role through telehealth. Clin J Oncol Nurs.

[51] Mariano MCO, Maniego JCM, Manila HLMD, Mapanoo RCC, Maquiran KMA, Macindo JRB (2018). Social media use profile, social skills, and nurse-patient interaction among registered nurses in tertiary hospitals: a structural equation model analysis. Int J Nurs Stud.

[52] Pennington M, Ring H, Howlett J, Smith C, Redley M, Murphy C (2019). The impact of an epilepsy nurse competency framework on the costs of supporting adults with epilepsy and intellectual disability: findings from the EpAID study. J Intellect Disabil Res.

[53] Gonzalez-Vega MP (2020). Experience of primary care nurses involved in the comprehensive care model. Rev Salud Publica (Bogota).

[54] Morelato CS, Dorneles LL, Martins VDP, Goés F, Viana AL, Brunello MEF (2021). Receiving spontaneous demand in primary care: nurses' learning needs. Rev Bras Enferm.

[55] Molina-Mula J, Gallo-Estrada J, Perelló-Campaner C (2017). Impact of interprofessional relationships from nurses' perspective on the decision-making capacity of patients in a clinical setting. Int J Environ Res Public Health.

[56] Chouinard V, Contandriopoulos D, Perroux M, Larouche C (2017). Supporting nurse practitioners' practice in primary healthcare settings: a three-level qualitative model. BMC Health Serv Res.

[57] Pype P, Mertens F, Helewaut F, Krystallidou D (2018). Healthcare teams as complex adaptive systems: understanding team behaviour through team members' perception of interpersonal interaction. BMC Health Serv Res.

[58] Toh LS, Lai PSM, Othman S, Wong KT, Low BY, Anderson C (2017). An analysis of inter-professional collaboration in osteoporosis screening at a primary care level using the D'Amour model. Res Social Adm Pharm.

[59] Skinner MS, Veenstra M, Sogstad M (2021). Nurses' assessments of horizontal collaboration in municipal health and care services for older adults: a cross-sectional study. Res Nurs Health.

[60] Tlili MA, Aouicha W, Ben Dhiab M, Mallouli M (2020). Assessment of nurses' patient safety culture in 30 primary health-care centres in Tunisia. East Mediterr Health J.

